# An Integrated Quasi-Zero-Stiffness Mechanism with Arrayed Piezoelectric Cantilevers for Low-Frequency Vibration Isolation and Broadband Energy Harvesting

**DOI:** 10.3390/s25165180

**Published:** 2025-08-20

**Authors:** Kangkang Guo, Anjie Sun, Junhai He

**Affiliations:** School of Measurement and Communication Engineering, Harbin University of Science and Technology, Harbin 150080, China; 18305508599@163.com (A.S.); 2320610213@stu.hrbust.edu.cn (J.H.)

**Keywords:** low-frequency vibration isolation, piezoelectric energy harvesting, quasi-zero-stiffness, multi-objective optimization, nonlinear dynamics, piezoelectric beam array

## Abstract

To address the collaborative demand for low-frequency vibration control and energy recovery, this paper proposes a dual-functional structure integrating low-frequency vibration isolation and broadband energy harvesting. The structure consists of two core components: one is a quasi-zero stiffness (QZS) vibration isolation module composed of a linkage-horizontal spring (negative stiffness) and a vertical spring; the other is an energy-harvesting component with an array of parameter-differentiated piezoelectric cantilever beams. Aiming at the conflict between the structural dynamic stiffness approaching zero and broadening the effective working range, this paper establishes a dual-objective optimization function based on the Pareto principle on the basis of static analysis and uses the grid search method combined with actual working conditions to determine the optimal parameter combination. By establishing a multi-degree-of-freedom electromechanical coupling model, the harmonic balance method is used to derive analytical solutions, which are then verified by numerical simulations. The influence laws of external excitations and system parameters on vibration isolation and energy-harvesting performance are quantitatively analyzed. The results show that the optimized structure has an initial vibration isolation frequency below 2 Hz, with a vibration isolation rate exceeding 60% in the 3 to 5 Hz ultra-low frequency range and a minimum transmissibility of the order of 10^−2^ (vibration isolation rate > 98%). The parameter-differentiated piezoelectric array effectively broadens the energy-harvesting frequency band, which coincides with the vibration isolation range. Synergistic optimization of both performances can be achieved by adjusting system damping, parameters of piezoelectric vibrators, and load resistance. This study provides a theoretical reference for the integrated design of low-frequency vibration control and energy recovery, and its engineering implementation requires further experimental verification.

## 1. Introduction

Vibration is widely present in industrial and daily life scenarios. On one hand, vibration, especially low-frequency vibration, can interfere with sensor accuracy, accelerate device aging, shorten equipment lifespan, and even induce structural resonance leading to system failure [1]. On the other hand, as a sensitive indicator of system states (e.g., structural damage), the efficient analysis and feature extraction of vibration signals are crucial for realizing intelligent structural health monitoring [2]. Meanwhile, as a form of energy, harvesting ambient vibrational energy has attracted significant attention due to its practical prospects in enabling self-powered wireless sensor network nodes and low-power electronic devices. Based on the dual attributes of vibration, being both a potential source of damage and a valuable information source and utilizable energy source, extensive research on vibration control and utilization has been stimulated among scholars [3,4,5].

Vibration control problems are generally categorized into two types: one where vibration originates from the load, with the goal of attenuating its transmission to the foundation (base or main structure), and the other where vibration comes from the foundation, aiming to suppress its transmission to the load [6]. Traditional linear vibration isolation systems struggle to ensure effective low-frequency vibration isolation while achieving high load-bearing capacity. This inherent limitation severely restricts their application in low-frequency vibration control. Quasi-zero stiffness (QZS) vibration isolation, characterized by high static stiffness and low dynamic stiffness, well compensates for this deficiency. QZS is typically realized through the parallel combination of positive and negative stiffness, with the construction of the negative stiffness mechanism being the core of the design. Common negative stiffness mechanisms include inclined spring-type [7], pre-buckled beam-type [8], cam-roller-spring mechanisms [9], and magnetic structures [10], as well as novel origami-based [11] and bionics-based [12,13] mechanisms.

In terms of vibration utilization, vibration energy harvesting is undoubtedly one of the research hotspots in recent years. Prevalent conversion mechanisms currently include piezoelectric [14], electrostatic [15], electromagnetic [16], triboelectric [17], and magnetostrictive [18] types. Among these, the piezoelectric type offers significant advantages such as flexible design, no electromagnetic interference, high power density, and ease of miniaturization. Many scholars have focused on broadening the energy-harvesting bandwidth, enhancing environmental adaptability, and improving energy conversion efficiency [19]. To this end, approaches such as optimizing the shape of elastic base beams [20], incorporating functionally graded intelligent composite materials [21], arranging piezoelectric beam arrays [22], and adopting nonlinear structures (e.g., bistable, multistable [23] based on non-resonant principles and QZS [24] structures) have been proposed.

In recent years, researchers have begun to design structures integrating both energy-harvesting and vibration isolation functions. Davis et al. [25] utilized the geometrically nonlinear stiffness of Euler-buckled columns to achieve QZS characteristics and attached piezoelectric patches in high-strain regions for an integrated vibration isolation and energy-harvesting design. Mofidian et al. [26] developed an electromagnetic induction-based dual-purpose device that not only achieves vibration isolation but also provides partial damping through the energy-harvesting process. Diala et al. [27] designed a device incorporating magnets, planar springs, coils, and air holes to realize integrated electromagnetic energy harvesting and vibration isolation. Yang et al. [28] developed a nonlinear mechanical oscillator with multistable characteristics, which exhibits excellent vibration isolation and energy-harvesting performance under low-intensity random or harmonic excitations. Zou et al. [29] created a device with customizable nonlinear forces to achieve dual functions of energy harvesting and vibration isolation. Lu et al. [30] designed an electromagnetic Stewart platform with high static and low dynamic stiffness for vibration isolation and energy harvesting. Cao et al. [31] proposed a low-frequency piezoelectric energy harvester integrated with a negative stiffness vibration isolator. Liu et al. [32] developed a QZS device based on piezoelectric buckled beams for vibration isolation and energy harvesting. However, these devices share a common issue: the effective energy-harvesting frequency range does not overlap with the vibration isolation range, preventing the two functions from operating simultaneously.

To achieve simultaneous dual functions, Lu et al. [33] and Li et al. [34] proposed dual-functional metamaterials for this integration. Wang et al. [35] achieved QZS by paralleling a coil spring with a magnetic negative stiffness mechanism, integrating piezoelectric cantilever beams and electromagnetic coils to synergistically enhance energy output through vibrational displacement and magnetic flux changes. Liu et al. [36] realized QZS via a cam-roller-spring mechanism, with multiple radial piezoelectric cantilever beams mounted on it; their analysis showed the effective energy-harvesting range falls within the vibration isolation range. Banerjee et al. [37] proposed a 3D superstructure with QZS characteristics, incorporating radially distributed cantilever beams to achieve dual functions. Tuo et al. [38] developed a QZS-based dual-functional mechanism using a piezoelectric buckled beam structure to meet vibration isolation and power supply requirements in automotive seat suspensions. Fang et al. [39] proposed a device coupling a cam-roller-spring mechanism with a nonlinear monostable energy harvester, adaptable to time-varying broadband vibrations for scenarios like intelligent floor tile systems. The aforementioned exploratory efforts in the field of synergistic dual-function vibration isolation and energy harvesting have laid a solid foundation for the development of this domain. Most existing devices are primarily designed for scenarios where vibration excitation originates from the load; however, in practical engineering applications, cases where vibration originates from the foundation and its transmission to the load needs to be suppressed are equally common and critical. Additionally, as shown in references [36,37], the optimal frequency band for energy harvesting (i.e., the resonance range of piezoelectric vibrators) is accompanied by an increase in transmissibility, which implies a degradation in vibration isolation performance. Meanwhile, piezoelectric cantilever beam arrays of a single size have a narrow resonance frequency band, making it difficult to match the broadband characteristics of environmental vibrations. Therefore, how to broaden the effective operating frequency band and achieve a balance between vibration isolation and energy-harvesting performance remains an urgent problem to be solved.

Through a comprehensive review and analysis of existing literature, it is found that the vast majority of studies utilize statics analysis to derive the parameter conditions for the system to exhibit quasi-zero stiffness (QZS) characteristics by ensuring that the minimum stiffness at the static equilibrium position is zero [40,41]. However, ensuring the minimum dynamic stiffness is zero is only one of the objectives. To effectively adapt to ultra-low frequency and wide amplitude range excitations, it is not only required that the mean dynamic stiffness in the quasi-zero region is as close to zero as possible, but also necessary to broaden the displacement range corresponding to the static equilibrium position, i.e., to expand the effective operating range of QZS. Notably, there is often a certain conflict between these two objectives: Typically, when striving for the minimum stiffness to be closer to zero, the effective operating range of the system may be compressed. Conversely, broadening the operating range may lead to an increased deviation of the minimum stiffness from zero. This mutual influence becomes more prominent when the structural dynamic stiffness is determined by multiple parameters. Therefore, it is difficult to determine the optimal parameter combination solely through statics analysis, and conducting multi-objective optimization to identify the optimal parameter combination is significantly necessary. It is worth noting that, in recent years, solutions based on optimization algorithms have made significant progress. For example, in the field of structural damage identification, the integration of vibration analysis and optimization algorithms has effectively improved prediction accuracy and efficiency [42,43]. This provides a new approach for obtaining optimal parameters in this domain.

To address this, this paper designs a dual-function structure for the typical working condition where vibration excitation originates from the foundation and vibration transmission to the load needs to be suppressed. The structure combines a quasi-zero stiffness (QZS) mechanism, formed by a link-horizontal spring negative stiffness structure and a vertical spring, with a piezoelectric cantilever beam array featuring parameter-differentiated design. Regarding the aforementioned dual-objective conflict between minimizing stiffness to approach zero and broadening the operating range, a dual-objective function is established based on the Pareto optimization principle, building upon static analysis. The optimal parameter combination is solved using the grid search method in conjunction with actual working conditions. Through a combination of analytical and numerical simulation methods, the influence laws of external excitation conditions and system parameters on vibration isolation and energy-harvesting performance are quantitatively analyzed. This verifies the effectiveness of the structure in achieving a synergistic optimization of ultra-low-frequency vibration isolation and broadband energy harvesting.

## 2. Structure Design and Parameter Optimization

### 2.1. Conceptual Design

**Figure 1:**
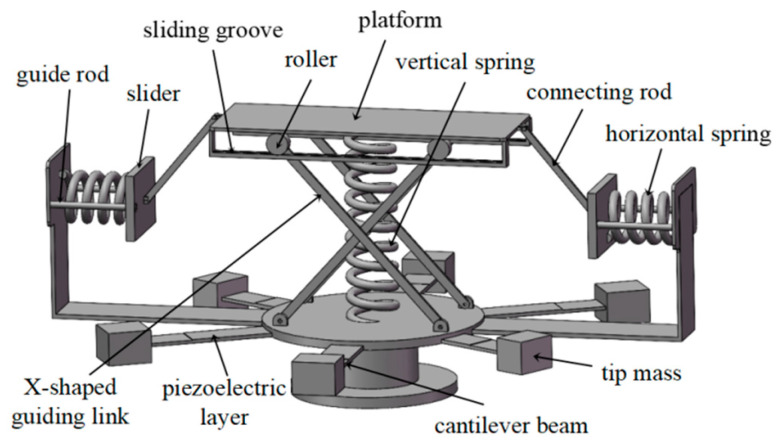
Physical model of the dual-functional structure.

The physical model of the proposed dual-functional structure is shown in Figure 1. This device consists of a QZS vibration isolation mechanism and radially distributed, arrayed piezoelectric cantilever beam energy-harvesting oscillators, integrating the dual functions of vibration isolation and energy harvesting. The vibration isolation structure comprises a vertical spring, a linkage-spring negative stiffness mechanism, and a motion guiding mechanism. The upper and lower ends of the vertical spring are fixedly connected to the load platform and the base platform, respectively, providing positive stiffness to support the isolated load. The negative stiffness mechanism consists of two symmetrically arranged connecting rods, two sliders, two horizontal springs, and four horizontal guide rods. Both ends of the horizontal springs are rigidly connected to the sliders and the structural constraint surfaces. The two ends of the connecting rods are rigidly fixed to the sliders and the load platform via high-strength bolts, ensuring no elastic deformation occurs when the load gravity is transmitted through the connecting rods. The sliders are inserted with four symmetrically distributed guide rods. The installation of all four guide rods must maintain coplanarity ≤ 0.01 mm/m and parallelism ≤ 0.02 mm/m. Friction is reduced through clearance fitting (tolerance controlled within 0.02 mm) and lubricating coatings, enabling the sliders to slide only in the horizontal direction. The motion guiding mechanism is composed of X-shaped guide links, sliding grooves, and rollers. The rollers form a rolling fit with the sliding grooves via bushings and are restricted to move only in the horizontal direction. Under the above constraints, the load platform and the isolated load can only move in the vertical direction. In the no-load state, the linkage forms an initial angle with the horizontal direction. When the platform generates vertical displacement, the horizontal spring-linkage mechanism produces negative stiffness through geometric nonlinearity: after the horizontal spring is compressed, its elastic force is converted into a vertical component force via the linkage, and the rate of change of this vertical component force with displacement is negative. By paralleling the positive stiffness of the vertical spring with the negative stiffness of the horizontal spring-linkage mechanism, the QZS characteristic can be achieved under specific parameter conditions, thereby blocking the transmission of foundation vibration to the load.

The piezoelectric energy-harvesting system consists of multiple groups of piezoelectric cantilever beam oscillators with different parameters. Each group contains several identical oscillators; oscillators within a group are symmetrically arranged, and oscillators between groups are radially distributed (as exemplified in the diagram and Section 4’s analysis, with three groups, each containing 2 identical oscillators). The root of the elastic base beam is fixedly connected to the base platform, and a lumped mass is attached to its free end. Piezoelectric patches are bonded to the root of the elastic base beam, with metal electrodes arranged on their upper and lower surfaces. The electrodes are connected in parallel to rectifier circuits, energy storage circuits, etc. (simplified as a load resistor in this paper). The entire system is subjected to base excitation.

### 2.2. Stiffness Analysis of QZS Structure

**Figure 2:**
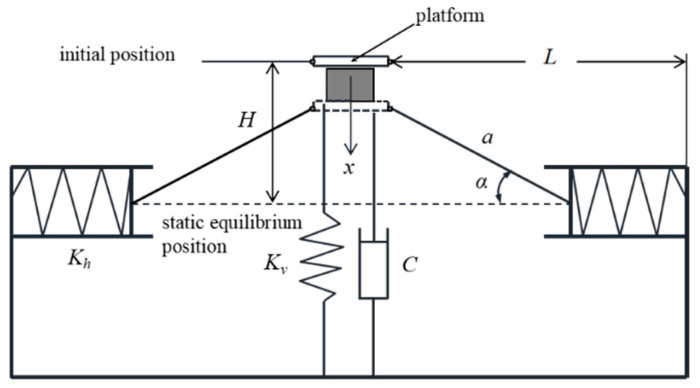
Schematic diagram of quasi-zero stiffness support.

As shown in the Figure 2, the two linkages are set to have a length of *a*, with their two ends hinged to the sliders and the loading platform. The horizontal springs and sliders form a low-friction transmission chain, where the friction coefficient is negligible, and their stiffness is uniformly *K_h_*; the stiffness of the vertical spring is *K_v_*. Taking the system in its initial position (i.e., under no-load condition) as the reference point, we let *H* be the distance from the loading platform to the static equilibrium position at this time, the initial length of the symmetrically arranged horizontal springs be *L_h_*_0_, and the distance between the isolated payload and the constraint surfaces on both sides be *L*. After applying a vertical load, the dynamic displacement of the mass relative to the initial position is denoted by *x*, and the angle between the linkage axis and the central axis of the horizontal spring is denoted by *α*. During the operation of the mechanism, the horizontal springs are compressed, with the compression amount denoted by ∆*L*; the pre-compression amount of the horizontal springs at the static equilibrium position is Δ*L*_0_.

Taking the position under the no-load condition as the reference point, after applying a downward force, the dynamic displacement is *x*. From the geometric relationship, it follows that

(1) $$\sin\alpha =\frac{H-x}{a}$$

(2) $$\cos\alpha =\frac{L-\left(L_{h0}-\Delta L\right)}{a}$$

(3) $$\Delta L=\sqrt{a^{2}-{\left(H-x\right)}^{2}}-\sqrt{a^{2}-H^{2}}$$

The vertical component force of the negative stiffness mechanism can be obtained as

(4) $$F_{h}(x)=2K_{h}\Delta L\cdot \frac{H-x}{L-\left(L_{h0}-\Delta L\right)}=2K_{h}\Delta L\cdot \frac{H-x}{\sqrt{a^{2}-{\left(H-x\right)}^{2}}}$$

The elastic force of the vertical spring is

(5) $$F_{v}(x)=K_{v}x$$

When a load force is applied and the static equilibrium position is reached, the total restoring force of the structure should be equal in magnitude and opposite in direction to the load force, and thus

(6) $$F(x)=2K_{h}\Delta L\cdot \frac{H-x}{\sqrt{a^{2}-{\left(H-x\right)}^{2}}}+K_{v}x$$

The stiffness is obtained as

(7) $$K(x)=\frac{dF(x)}{dx}=-2K_{h}\Delta L\cdot \frac{a^{2}}{{\left[a^{2}-{\left(H-x\right)}^{2}\right]}^{3/2}}+K_{v}$$

By introducing the following non-dimensional quantities

(8) $$\eta =\frac{x}{a};\;h=\frac{H}{a};\;k_{h}=\frac{K_{h}}{K_{v}};\;\delta =\frac{\Delta L}{a};\;\delta_{0}=\frac{\Delta L_{0}}{a};\;f=\frac{F}{F_{0}}=\frac{F}{K_{v}a}$$

the dimensionless force–displacement relationship is expressed as

(9) $$f(\eta )=2k_{h}\delta \cdot \frac{h-\eta }{\sqrt{1-{\left(h-\eta \right)}^{2}}}+\eta$$

The dimensionless stiffness is derived by differentiating the dimensionless restoring force with respect to the dimensionless displacement, yielding the following:

(10) $$k_{\mathrm{stiff}}(\eta )=-2k_{h}\delta \cdot \frac{1}{{\left[1-{\left(h-\eta \right)}^{2}\right]}^{3/2}}+1$$

**Figure 3:**
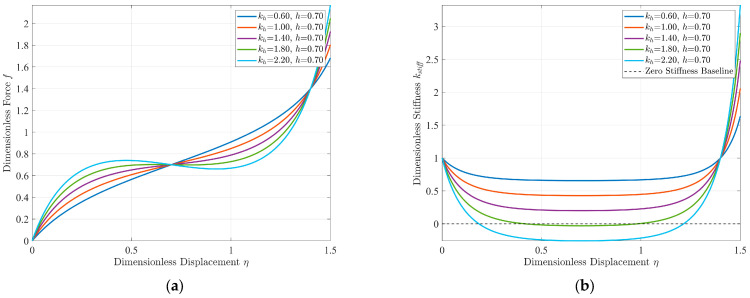
(**a**) Dimensionless force–displacement characteristic curves; (**b**) dimensionless stiffness– displacement characteristic curves under different stiffness ratios (*h* = 0.7).

**Figure 4:**
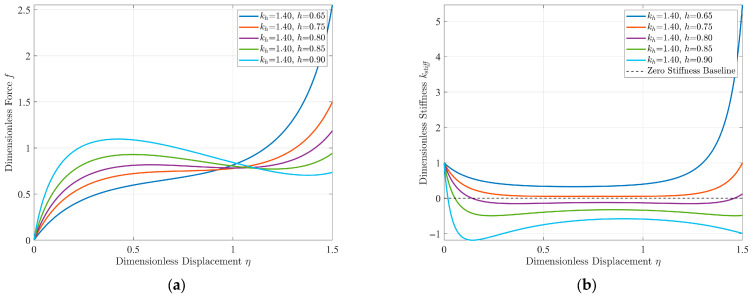
(**a**) Dimensionless force–displacement characteristic curves and (**b**) dimensionless stiffness–displacement characteristic curves under different no-load distances (*k_h_* = 1.4).

Based on Equations (9) and (10), the dimensionless force–displacement and dimensionless stiffness–displacement characteristic curves of the quasi-zero stiffness vibration isolation structure can be obtained, respectively. It is not difficult to see from the equations that the parameters affecting the dynamic stiffness mainly include the ratio of horizontal stiffness to vertical stiffness *k_h_*, the dimensionless no-load distance *h*, and the dimensionless compression amount of the horizontal spring *δ*_0_. Among them, the dimensionless no-load distance and the dimensionless compression amount of the horizontal spring are not independent of each other. From a practical perspective, this paper takes the stiffness ratio *k_h_* and the dimensionless no-load distance *h* as the core design parameters. As shown in Figure 3 and Figure 4, they are the comparison diagrams of the stiffness–displacement characteristic curves of the structure under different design parameters.

It can be seen from Figure 3 that, as the stiffness ratio increases continuously, the dynamic stiffness of the structure gradually decreases, changing from positive to quasi-zero and then to negative. Moreover, the displacement span corresponding to the minimum dynamic stiffness region remains unaffected. As shown in Figure 4, with the continuous increase in the dimensionless no-load distance, the dynamic stiffness of the structure also decreases gradually, changing from positive to quasi-zero and then to negative, but the displacement span corresponding to the minimum dynamic stiffness region increases accordingly.

### 2.3. Multi-Objective Optimization and Optimal Parameter Determination

The core requirement for the quasi-zero stiffness characteristic is to achieve an equivalent stiffness approaching zero in the neighborhood of the equilibrium position through the parallel optimization of the vertical spring and the linkage-horizontal spring negative stiffness compensation mechanism. This extends the lower limit of the effective vibration isolation frequency to a lower frequency band, enabling effective isolation of low-frequency vibrations. Meanwhile, it is expected that the structure can maintain the near-zero stiffness characteristic within a larger displacement range, i.e., maintain a balanced state where the superposition of positive and negative stiffness approaches zero over a wide displacement interval. This avoids a sudden increase in stiffness due to displacement exceeding the quasi-zero region, which would degrade the vibration isolation performance, thereby improving the performance stability of the vibration isolation system and its adaptability to external excitation conditions.

To find the optimal parameter combination, the quasi-zero region is defined as the displacement interval $\left[\eta_{\mathrm{low}},\eta_{\mathrm{high}}\right]$ around the static equilibrium position. We let *ε* be the stiffness threshold (set to *ε* = 0.1). Within this interval, *N* discrete displacement samples are uniformly selected. The interval is considered to exhibit the desired quasi-zero stiffness characteristic if the stiffness at all sampling points satisfies $\left|k_{\mathrm{stiff}}(\eta_{i})\right|\leq \varepsilon$, $\eta_{i}\in \left[\eta_{\mathrm{low}},\eta_{\mathrm{high}}\right]\left(i=1,2,\ldots ,N\right)$. Based on this criterion, the following multi-objective optimization functions are formulated as

(11) $$J_{1}(k_{h},h)=\frac{1}{N}\sum_{i=1}^{N}\left|k_{\mathrm{stiff}}(\eta_{i};k_{h},h)\right|=\frac{1}{N}\sum_{i=1}^{N}\left|\frac{-2k_{h}\delta }{{\left[1-{\left(h-\eta_{i}\right)}^{2}\right]}^{3/2}}+1\right|$$

(12) $$J_{2}(k_{h},h)=\eta_{\mathrm{high}}-\eta_{\mathrm{low}}=max\{\eta \left|\left|k_{\mathrm{stiff}}\;(\eta ;k_{h},h)\right|\leq \varepsilon \right.\}-min\{\eta \left|\left|k_{\mathrm{stiff}}\;(\eta ;k_{h},h)\right|\leq \varepsilon \right.\}$$

Based on the above analysis, the optimization objectives are determined as $\min J_{1}(k_{h},h)$ and $\max\;J_{2}(k_{h},h)$. Meanwhile, considering the structural design constraints and the feasibility of parameters, the design constraints and parameter value ranges are specified as follows:

(13) $$\left\{\begin{array}{l} 1-{\left(h-\eta \right)}^{2}>0\\ \sum_{i=1}^{N}\;I\left(\left|k_{\mathrm{stiff}}(\eta_{i};k_{h},h)\right|\leq \varepsilon \right)\geq 2\\ 0<h<1\\ 0\leq \eta \leq 1.5\\ 1\leq k_{h}\leq 3 \end{array}\right.$$

**Figure 5:**
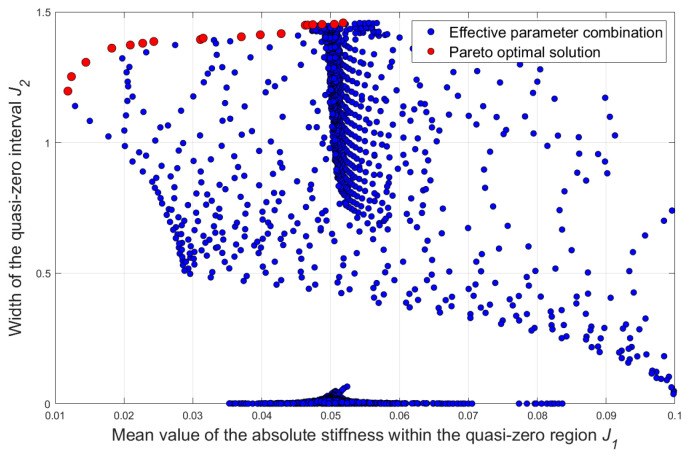
Scatter plot of optimization objectives via grid search method: non-optimal samples (blue points) and Pareto frontier solutions (red points).

**Figure 6:**
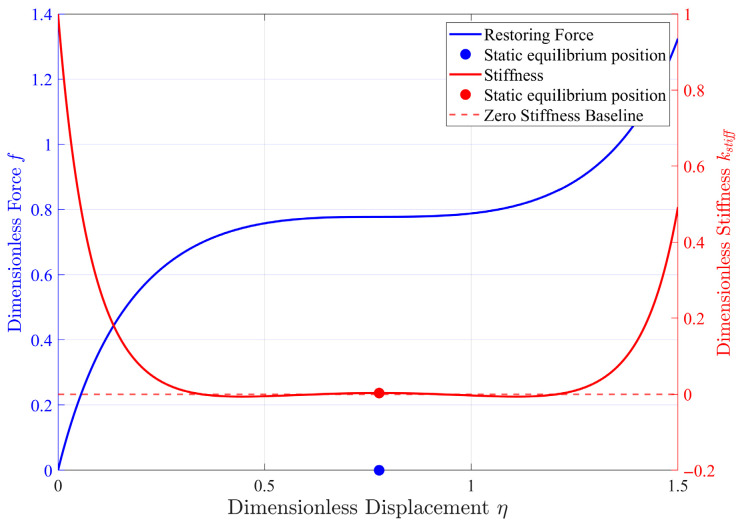
Dimensionless force–displacement curve (left y-axis) and dimensionless stiffness–displacement characteristic curve (right y-axis) of the optimized system.

A scatter plot of the width of the quasi-zero stiffness region and the mean value of the absolute stiffness within the region based on the grid search method is shown in Figure 5. The red scatter points represent the Pareto optimal solutions obtained through the grid search. In the trade-off of the “region width-stiffness mean value” objectives, these solutions form the frontier of the optimal solution set for multi-objective optimization. Through this plot, the correlation between the width of the quasi-zero stiffness region and the mean value of the absolute stiffness within the region can be intuitively observed. Subsequently, according to engineering requirements (such as emphasizing a wider quasi-zero region or a lower stiffness mean value), appropriate parameter combinations can be reasonably selected from the Pareto optimal solution set. In this paper, based on the characteristics of the Pareto frontier (with small variation in J_2_, large span in J_1_, and the left-side point being more advantageous as they can better balance “wide displacement” and “low stiffness” to adapt to working conditions), the corresponding optimal parameter combination ($k_{h}=1.3434,\;h=0.7775,\;\delta_{0}=0.371$) is selected. For this combination, the mean stiffness corresponding to the QZS region is *J*_1_ = 0.0119, and the width of the QZS interval is *J*_2_ = 1.1968. And the force–displacement and stiffness–displacement curves under these parameters are drawn, as shown in Figure 6. As can be seen from the figure, under the corresponding optimal parameter combination, the dynamic stiffness of the structure at the static equilibrium position is approximately zero, and it remains approximately zero within a relatively wide displacement interval. At this time, if the system vibrates within the corresponding interval range around the static equilibrium position under the action of external excitation, the dynamic stiffness of the system is always approximately zero. Meanwhile, the positive stiffness of the vertically arranged spring ensures a high bearing capacity when the structure is stationary at the equilibrium position, achieving the “high static and low dynamic” characteristic. According to the vibration isolation principle, this structure can achieve the purpose of low-frequency vibration isolation.

## 3. Dynamic Modeling and Analysis

In this section, the dynamic model of the dual-function system is established by treating both the linkage-spring quasi-zero stiffness structure and the piezoelectric cantilever beam as single-degree-of-freedom systems. Furthermore, the Harmonic Balance Method (HBM) is employed to analyze the steady-state periodic response characteristics of the system, yielding the displacement transmissibility and average output power.

### 3.1. Dynamic Model

**Figure 7:**
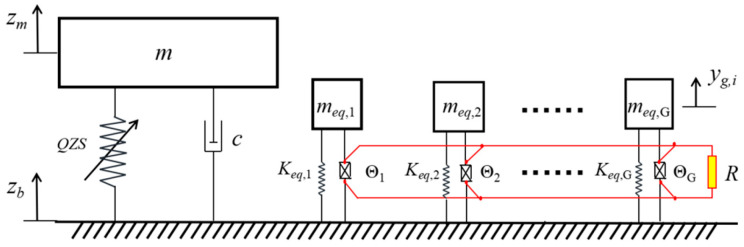
Simplified dynamic model of the proposed structure.

The simplified dynamic model of the entire structure is shown in Figure 7. Under base excitation, the quasi-zero stiffness vibration isolation structure vibrates in the vertical direction, while the piezoelectric cantilever beam energy harvester undergoes lateral vibration. The absolute displacements of the loading platform and the base are denoted by *z_m_* and *z_b_*, respectively, and the relative displacement of the platform is *z* = *z_m_* − *z_b_*. The absolute displacements of the piezoelectric cantilever beam vibrators with different parameters are denoted by *y_g_* (the subscript *g* denotes the vibrator group).

When the rated load is fixed on the platform, the platform will move down to the static equilibrium position, where the system has a dynamic stiffness close to zero, so as to achieve the design goal of low-frequency vibration isolation. Under base excitation, the load will vibrate around the equilibrium position. For this reason, the reference point is set at the static equilibrium position, and upward displacement is defined as positive. The dynamic equation of the QZS system can be established as Equation (14).

(14) $$m\ddot{z}+c\dot{z}+F_{QZS}(z)=-m{\ddot{z}}_{b}$$

Then we developed the electromechanical coupled relations of the piezoelectric cantilever beam with tip mass. The stress–strain relationship of the elastic matrix layer is given by

(15) $$T_{1}=c_{11}^{s}S_{1}$$

where *T*_1_ and *S*_1_ denote the mechanical stress and strain along the longitudinal direction, respectively, and $c_{11}^{s}$ is the substrate stiffness coefficient. For the piezoelectric patch, the constitutive relationship with piezoelectric effect can be expressed as

(16) $$\left\{\begin{array}{l} T_{1}=c_{11}^{E}S_{1}-e_{31}E_{3}\\ D_{3}=\varepsilon_{33}^{S}E_{3}+e_{31}S_{1} \end{array}\right.$$

where *E*_3_ and *D*_3_ are the electric field and displacement along direction 3, respectively, $c_{11}^{E}$ is the elastic stiffness at zero electric field, *e*_31_ is the piezoelectric coefficient, and $\varepsilon_{33}^{S}$ represents the permittivity constant at zero strain.

Using the first-order vibration mode as the shape function and applying the principle of virtual work, the electromechanical coupling equations for the arrayed piezoelectric cantilever beam energy-harvesting structure are derived as

(17) $$m_{eq,g}{\ddot{y}}_{g,i}+K_{eq,g}y_{g,i}+\Theta_{g}v=-m_{eq,g}{\ddot{z}}_{b}$$

(18) $$I_{g,i}=-\Theta_{g}{\dot{y}}_{g,i}+C_{p,g}\dot{v}$$

where

(19) $$m_{eq,g}=m_{t,g}+\rho_{g}{\int_{0}^{L_{b,g}}\left(\phi_{g}(s)\right)}^{2}ds$$

(20) $$K_{eq,g}=c_{11}^{s}\int_{A_{b,g}}{\bar{z}}^{2}dA_{b,g}\int_{0}^{L}{\left(\phi_{g}^{\prime \prime }(s)\right)}^{2}ds+\frac{1}{4}c_{11}^{E}bh_{p,g}h_{b,g}^{2}\int_{0}^{L_{p.g}}{\left(\phi_{g}^{\prime \prime }(s)\right)}^{2}ds$$

(21) $$\Theta_{g}=\frac{1}{2}e_{31}b_{g}h_{b,g}\phi^{\prime }\left(L_{p,g}\right)$$

(22) $$C_{p,g}=\frac{\varepsilon_{33}^{S}b_{g}L_{p,g}}{h_{p,g}}$$

where *g* = 1, 2, …, *G* denote the vibrator groups, *i* = 1, 2, …, *n*_g_ denote the numbers of vibrators within each group, *m_eq_* and *K_eq_* are the equivalent mass and equivalent stiffness, respectively, and Θ and *C_p_* are the electromechanical coupling coefficient and the capacitance of the piezoelectric element. *m_t_* is the tip mass, and *ρ* is the mass per unit length of the beam. *b* and *L_p_* are the width and length of the piezoelectric layer. *L_b_* is the length of the beam. *h_p_* is the thickness of the piezoelectric layer. *h_b_* is the thickness of the beam, and *A_b_* is the cross section of the beam. $\bar{z}$ is the transverse coordinate from the neutral surface. *ϕ*(*s*) denotes the normalized shape function [44]

(23) $$\phi (s)=\frac{\left[\sin(qL_{b})+\sinh(qL_{b})][\cos(qs)-\cosh(qs)]-[\cos(qL_{b})+\cosh(qL_{b})][\sin(qs)-\sinh(qs)\right]}{2[\cos(qL_{b})\sinh(qL_{b})-\sin(qL_{b})\cosh(qL_{b})]}$$

wherein *s* is the longitudinal coordinate of the cantilever beam, and *q* is solved from the following characteristic equation:

(24) $$\frac{m_{t}}{\rho L_{b}}(qL_{b})[\cos(qL_{b})\sinh(qL_{b})-\sin(qL_{b})\cosh(qL_{b})]+\cos(qL_{b})\cosh(qL_{b})+1=0$$

Considering that there are *G* × *n_g_* piezoelectric cantilever beams, one can obtain

(25) $$-\frac{v}{R}=\sum_{g=1}^{G}\sum_{i=1}^{n_{g}}I_{i}=-\sum_{g=1}^{G}\sum_{i=1}^{n_{g}}\Theta_{g}{\dot{y}}_{g,i}+\left(\sum_{g=1}^{G}n_{g}C_{p,g}\right)\dot{v}$$

The electromechanical coupling equations of the entire system can be expressed as

(26) $$\left\{\begin{array}{l} m\ddot{z}+c\dot{z}+F_{QZS}(z)=-m{\ddot{z}}_{b}\\ m_{eq,g}{\ddot{y}}_{g,i}+K_{eq,g}y_{g,i}+\Theta_{g}v=-m_{eq,g}{\ddot{z}}_{b}\\ v/R+\left(\sum_{g=1}^{G}n_{g}C_{p,g}\right)\dot{v}-\sum_{g=1}^{G}\sum_{i=1}^{n_{g}}\Theta_{g}{\dot{y}}_{g,i}=0 \end{array}\right.$$

with the parameters defined as followed:

(27) $$\begin{array}{l} \mu_{g}=\frac{m_{eq,g}}{m};\kappa_{g}=\frac{K_{eq,g}}{K_{v}};\theta_{g}=\frac{\Theta_{g}}{\sqrt{K_{eq}^{\mathrm{ref}}C_{p}^{\mathrm{ref}}}};\omega_{0}=\sqrt{\frac{K_{v}}{m}};\zeta =\frac{c}{2m\omega_{0}};Z=\frac{z}{a}=\frac{z_{m}-z_{b}}{a};\\ Y_{g,i}=\frac{y_{g,i}}{a};Z_{b}=\frac{z_{b}}{a};\Omega =\frac{\omega }{\omega_{0}};\tau =\omega_{0}t;\vartheta =\frac{1}{RC_{p}^{\mathrm{ref}}\omega_{0}};u=\frac{v}{a\sqrt{K_{eq}^{\mathrm{ref}}/C_{p}^{\mathrm{ref}}}};f_{QZS}=\frac{F_{QZS}}{K_{v}a} \end{array}$$

Equation (25) can be rewritten in a dimensionless form

(28) $$\left\{\begin{array}{l} \ddot{Z}+2\zeta \dot{Z}+f_{QZS}(Z)=-{\ddot{Z}}_{b}\\ \mu_{g}{\ddot{Y}}_{g,i}+\kappa_{g}Y_{g,i}+\theta_{g}u+\mu_{g}{\ddot{Z}}_{b}=0\\ \vartheta u+\bar{C}\dot{u}-\sum_{g=1}^{G}n_{g}\theta_{g}{\dot{Y}}_{g}=0 \end{array}\right.(g=1,2,\cdots ,G;i=1,2,\cdots ,n_{g})$$

where $\bar{C}=\sum_{g=1}^{G}n_{g}\frac{C_{p,g}}{C_{p}^{\mathrm{ref}}}$.

**Figure 8:**
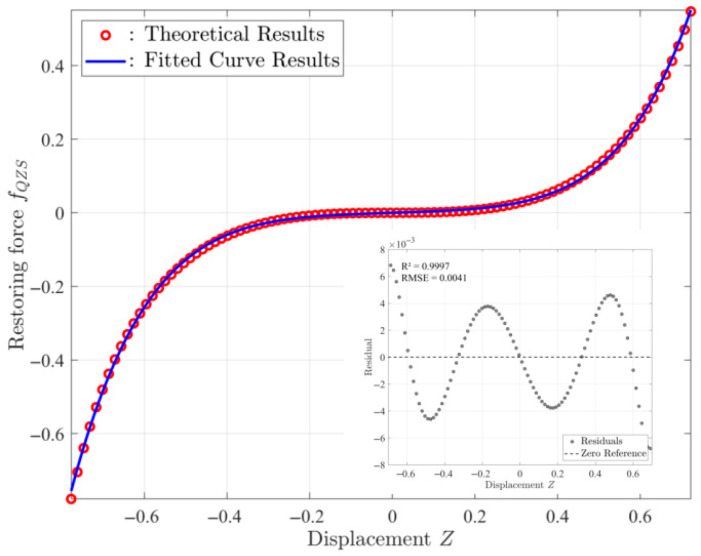
Theoretical and fitting curves of dimensionless restoring force with residual plot.

The dimensionless restoring force *f_QZS_* can be obtained by performing coordinate transformation on Equation (9). It is evident that Equation (9) contains an irrational fraction term, which brings inconvenience to the analytical analysis of dynamic response. Therefore, it is necessary to either perform a Taylor series expansion of the equation or fit the force–displacement characteristic curve after coordinate transformation to obtain an approximate polynomial for the force–displacement relationship. In this paper, a fifth-order polynomial model is used for fitting. The comparison between the fitting results and theoretical calculation results, along with residual analysis, is shown in Figure 8. The results indicate that the fitting results are in good agreement with the theoretical results, with a coefficient of determination *R*^2^ = 0.9997 and a root mean square error RMSE = 0.0041, demonstrating that the polynomial can accurately characterize the force–displacement relationship of the quasi-zero stiffness (QZS) system. The fitted fifth-order polynomial model is expressed as

(29) $$f_{QZS}(Z)\approx \lambda Z+\gamma Z^{3}+\beta Z^{5}=\omega_{n}^{2}Z+\gamma Z^{3}+\beta Z^{5}$$

Substituting Equation (29) into Equation (28), it can be written as

(30) $$\left\{\begin{array}{l} \ddot{Z}+2\zeta \dot{Z}+\omega_{n}^{2}Z+\gamma Z^{3}+\beta Z^{5}=-{\ddot{Z}}_{b}\\ \mu_{g}{\ddot{Y}}_{g,i}+\kappa_{g}Y_{g,i}+\theta_{g}u+\mu_{g}{\ddot{Z}}_{b}=0\\ \vartheta u+\bar{C}\dot{u}-\sum_{g=1}^{G}n_{g}\theta_{g}{\dot{Y}}_{g}=0 \end{array}\right.(g=1,2,\cdots ,G;i=1,2,\cdots ,n_{g})$$

### 3.2. HBM-Based Solution

Let the base excitation be the following:

(31) $$Z_{b}=a_{b}\cos\Omega \tau =\frac{a_{b}}{2}e^{j\Omega \tau }+cc$$

where *j* is the imaginary unit ($j=\sqrt{-1}$) and *cc* is the complex conjugate. Let the steady-state response solution of the Equation (30) be the following:

(32) $$\left\{\begin{array}{c} Z(\tau )=a_{0}\cos\left(\Omega \tau +\varphi_{1}\right)=\frac{A}{2}e^{j\Omega \tau }+cc\\ Y_{g}(\tau )=b_{0g}\cos\left(\Omega \tau +\varphi_{2g}\right)=\frac{B_{g}}{2}e^{j\Omega \tau }+cc\\ u(\tau )=c_{0}\cos\left(\Omega \tau +\varphi_{3}\right)=\frac{C}{2}e^{j\Omega \tau }+cc \end{array}\right.(g=1,2,\cdots ,G)$$

where *a*_0_, *b*_0*g*_, and *c*_0_ are the response amplitudes and $\varphi_{1}$, $\varphi_{2}$, and $\varphi_{3}$ are the phace. Additionally, *A*, *B_g_*, and *C* are complex numbers, defined as

(33) $$\left\{\begin{array}{l} A=a_{0}e^{j\varphi_{1}}\\ B_{g}=b_{0g}e^{j\varphi_{2g}}\\ C=c_{0}e^{j\varphi_{3}} \end{array}\right.$$

Substituting Equations (31) and (32) into Equation (30), the following equations can be obtained as

(34) $$\left(-\Omega^{2}+j\Omega \cdot 2\zeta +\omega_{n}^{2}\right)A+\frac{3}{4}\gamma {\left|A\right|}^{2}A+\frac{5}{8}\beta {\left|A\right|}^{4}A=a_{b}\Omega^{2}$$

(35) $$\left(\kappa_{g}-\mu_{g}\Omega^{2}\right)B_{g}+\theta_{g}C-\mu_{g}a_{b}\Omega^{2}=0(g=1,2,\cdots ,G)$$

(36) $$\left(\vartheta +j\Omega \bar{C}\right)C-j\Omega \sum_{g=1}^{G}n_{g}\theta_{g}B_{g}=0$$

Combining Equations (35) and (36) gives

(37) $$C=\frac{j\Omega^{3}a_{b}\sum_{g=1}^{G}\frac{n_{g}\theta_{g}\mu_{g}}{\kappa_{g}-\mu_{g}\Omega^{2}}}{\vartheta +j\Omega \left(\bar{C}+\sum_{g=1}^{G}\frac{n_{g}\theta_{g}^{2}}{\kappa_{g}-\mu_{g}\Omega^{2}}\right)}$$

Considering $A=a_{0}e^{j\varphi_{1}}$ and $\tilde{A}=a_{0}e^{-j\varphi_{1}}$, substituting into Equation (34), and separating the real and imaginary parts yields the following:

(38) $$\begin{array}{l} \left(\omega_{n}^{2}-\Omega^{2}\right)a_{0}\cos\varphi_{1}-2\zeta \Omega a_{0}\sin\varphi_{1}+\frac{3}{4}\gamma a_{0}^{3}\cos\varphi_{1}+\frac{5}{8}\beta a_{0}^{5}\cos\varphi_{1}=a_{b}\Omega^{2}\\ (\omega_{n}^{2}-\Omega^{2})a_{0}\sin\varphi_{1}+2\zeta \Omega a_{0}\cos\varphi_{1}+\frac{3}{4}\gamma a_{0}^{3}\sin\varphi_{1}+\frac{5}{8}\beta a_{0}^{5}\sin\varphi_{1}=0 \end{array}$$

Squaring and adding yields

(39) $${\left[\left(\omega_{n}^{2}-\Omega^{2}\right)a_{0}+\frac{3}{4}\gamma a_{0}^{3}+\frac{5}{8}\beta a_{0}^{5}\right]}^{2}+{\left(2\zeta \Omega a_{0}\right)}^{2}={\left(a_{b}\Omega^{2}\right)}^{2}$$

Solving this equation yields the response amplitude of the payload. The absolute displacement of the platform can be expressed as

(40) $$Z_{m}=Z+Z_{b}=a_{0}\cos\left(\Omega \tau +\varphi_{1}\right)+a_{b}\cos\Omega \tau$$

Vibration isolation performance is quantified by the displacement transmissibility, defined as

(41) $$T_{d}=\frac{\left|Z_{m}\right|}{\left|Z_{b}\right|}=\frac{\sqrt{a_{0}^{2}+2a_{0}a_{b}\cos\varphi_{1}+a_{b}^{2}}}{a_{b}}=\frac{\sqrt{a_{0}^{2}\left(2\lambda -\Omega^{2}\right)+\frac{3}{2}\gamma a_{0}^{4}+\frac{5}{4}\beta a_{0}^{6}+a_{b}^{2}\Omega^{2}}}{a_{b}\Omega }$$

The output voltage amplitude is

(42) $$c_{0}=|C|=\left|\frac{j\Omega^{3}a_{b}\sum_{g=1}^{G}\frac{n_{g}\theta_{g}\mu_{g}}{\kappa_{g}-\mu_{g}\Omega^{2}}}{\vartheta +j\Omega \left(\bar{C}+\sum_{g=1}^{G}\frac{n_{g}\theta_{g}^{2}}{\kappa_{g}-\mu_{g}\Omega^{2}}\right)}\right|$$

Output power can be calculated by

(43) $$p(t)=\frac{{\left[v(t)\right]}^{2}}{R}$$

By introducing the dimensionless power $P=\frac{p}{K_{eq}^{\mathrm{ref}}\omega_{0}a^{2}}$ and leveraging the non-dimensional quantities (e.g., dimensionless time *τ*, dimensionless voltage *u* and dimensionless parameter *ϑ* defined in Equation (27)), the dimensionless power can be expressed as

(44) $$P(\tau )=\vartheta {\left[u(\tau )\right]}^{2}$$

For the sinusoidal alternating voltage, the average output power (dimensionless) across the load resistor is

(45) $$P_{a}=\frac{\omega }{2\pi }\int_{\tau }^{\tau +\frac{2\pi }{\omega }}P(\tau )\;d\tau =\frac{\vartheta {\left|C\right|}^{2}}{2}$$

## 4. Results and Discussions

**Figure 9:**
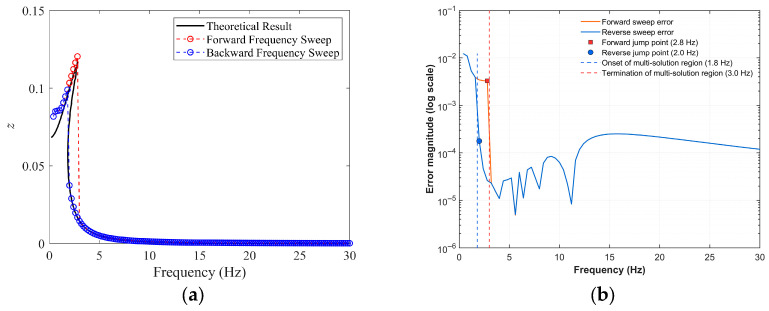
Comparison of response amplitude–excitation frequency curves between analytical and numerical simulation results with error analysis (*Z_e_* = 0.5 g; *ζ* = 0.05). (**a**) Curve comparison; (**b**) error analysis.

**Figure 10:**
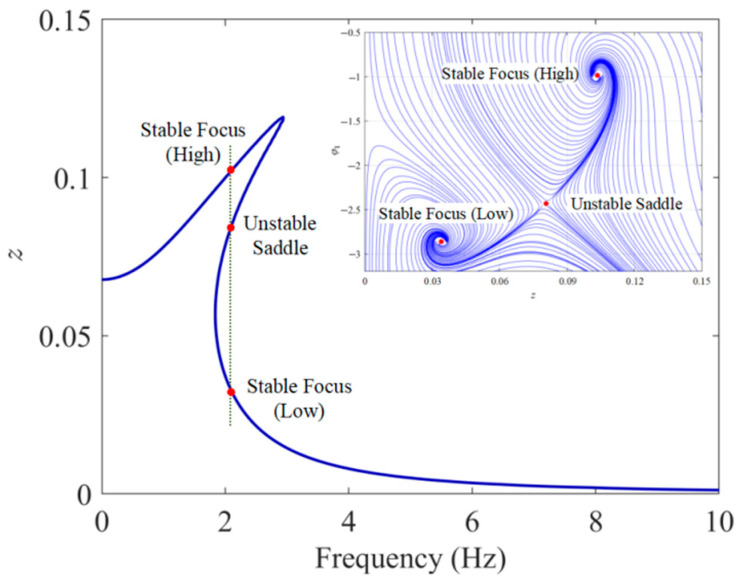
Multi-solution characteristics of steady-state responses near resonant frequency and phase trajectories in the (*a*_0_, *ϕ*_1_) plane (*Z_e_* = 0.5 g; *ζ* = 0.05).

To validate the effectiveness and accuracy of the harmonic balance method, the Runge–Kutta method was employed to numerically integrate the electromechanical coupling equations. The material and geometrical parameters of the dual-functional structure are tabulated in Table 1. Based on the given parameters, Figure 9 compares the analytical and numerical results under a base excitation amplitude of 0.5 g and conducts an error analysis, where *z* in the figure denotes the steady-state response amplitude of relative displacement. As can be seen from Figure 9a, the resonance peak of the analytical solution shifts to the right, exhibiting characteristics of multiple solutions and hardening nonlinearity, which corresponds to the jump phenomena observed in the upward and downward frequency sweep simulations. The error analysis results in Figure 9b show that in the ultra-low frequency range (within 1.4 Hz) and the multi-solution region is relatively large, with the maximum error value at the order of 10^−2^. After 3 Hz, the average error amplitude drops to the order of 10^−4^, and the overall agreement is good. Figure 10 further shows the multi-solution phenomenon of the steady-state response solutions near the dimensionless resonant frequency (*ω_n_* = 0.192) and the phase trajectories starting from different (*a*_0_ and $\varphi_{1}$) when the excitation frequency lies between the two jump frequencies. It is evident that, among these three solution branches, the middle solution branch is always unstable, corresponding to a saddle point, while the upper and lower solution branches are always stable, corresponding to focus. Which stable focus a solution is attracted to depends on the initial conditions, and this theoretically explains the jump phenomena in the forward and reverse sweep simulation experiments. From the perspective of broadening the vibration isolation operating frequency band, it is desirable for the system’s response solution to reside on the lower solution branch.

**Figure 11:**
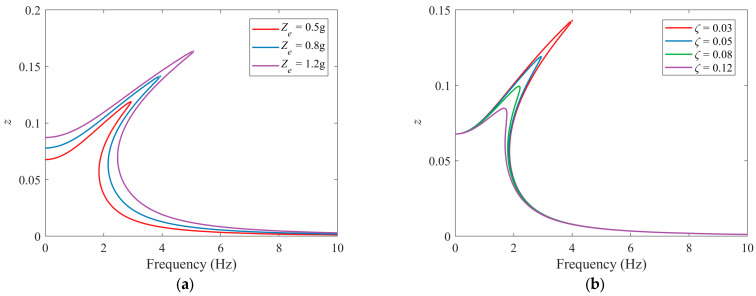
Response amplitude–excitation frequency curves under different values of (**a**) acceleration excitation amplitude *Z_e_* (*ζ* = 0.05) and (**b**) damping ratio *ζ* (*Z_e_* = 0.5 g).

Figure 11 illustrates the variation of steady-state amplitude–frequency response curves with respect to excitation amplitude *Z_e_* and damping ratio *ζ*. As shown in Figure 11a, increasing the excitation amplitude leads to a more pronounced rightward peak shift, suggesting enhanced nonlinear effects. However, the excitation amplitude has negligible influence on the high-frequency region (above 8 Hz). Figure 11b demonstrates that higher damping effectively suppresses the primary resonance peak. When the damping ratio *ζ* increases from 0.03 to 0.12, the resonance peak shift phenomenon is significantly mitigated.

**Figure 12:**
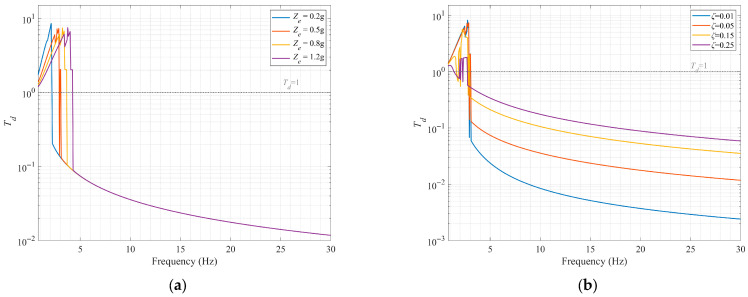
Absolute displacement transmissibility vs. excitation frequency under varied (**a**) excitation amplitudes *Z_e_* (*ζ* = 0.05) and (**b**) damping ratios *ζ* (*Z_e_* = 0.5 g).

Unlike the relationship between the steady-state response amplitude and the excitation frequency, which can be derived from the amplitude–frequency response equation (an algebraic equation), the relationship between the system’s absolute displacement transmissibility and the excitation frequency must be effectively solved by combining numerical calculations (such as the Newton iteration method). Figure 12 shows the curves of the relationship between absolute displacement transmissibility and excitation frequency under different excitation amplitudes and damping ratios, obtained by combining harmonic balance points and the Newton iteration method. Although the multi-solution phenomenon in the low-frequency region cannot be presented, the instability of solutions in this region can be observed. It can be seen from the Figure 12a that, as the excitation amplitude increases, the initial vibration isolation frequency shifts to the right, and the effective vibration isolation frequency band narrows. In Figure 12b, the variation of displacement transmissibility under different damping ratios is presented, with the excitation amplitude *Z_e_* set to 0.5 g. Changes in the damping ratio have almost no effect on the initial vibration isolation frequency. As the damping ratio *ζ* increases, the peak transmissibility decreases; however, the vibration isolation performance within the effective vibration isolation range deteriorates significantly. Taking 5 Hz as an example, the transmissibility is 0.024 when *ζ* is 0.01 and 0.339 when *ζ* is 0.25, representing a 31.6% decrease in vibration attenuation. Therefore, the consideration of damping in the vibration isolation system should be based on actual working conditions.

**Figure 13:**
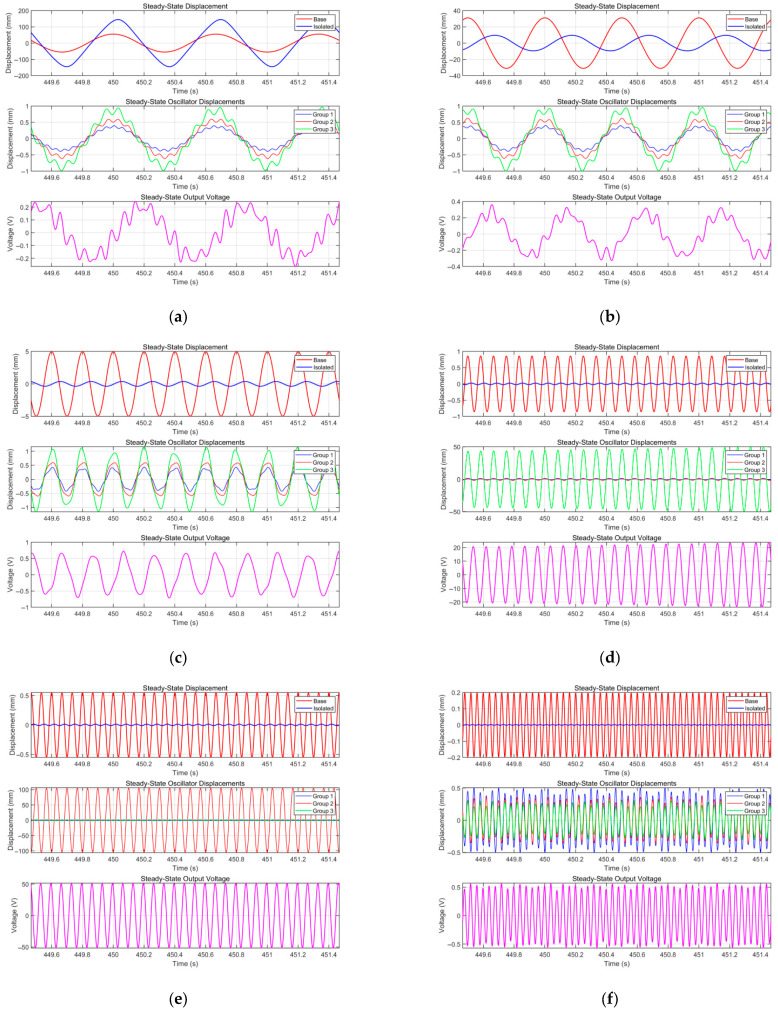
System response characteristics under different harmonic excitation frequencies (*Z_e_* = 0.5 g; *ζ* = 0.05; *R* = 450 kΩ): comparison of displacement time histories between the base and the isolated load (**top**); displacement time histories of three groups of piezoelectric cantilever oscillators with different sizes (**middle**); and voltage time-domain diagrams (**bottom**). (**a**) *ω* = 1.5 Hz, *T_d_* = 2.626, *v*_RMS_ = 0.150 V; (**b**) *ω* = 2 Hz, *T_d_* = 0.307, *v*_RMS_ = 0.191 V; (**c**) *ω* = 5 Hz, *T_d_* = 0.074, *v*_RMS_ = 0.443 V; (**d**) *ω* = 12 Hz, *T_d_* = 0.030, *v*_RMS_ = 16.507 V; (**e**) *ω* = 15 Hz, *T_d_* = 0.024, *v*_RMS_ = 36.459 V; (**f**) *ω* = 25 Hz, *T_d_* = 0.014, *v*_RMS_ = 0.376 V.

To clearly observe the influence of excitation frequency on steady-state response, Figure 13 presents vibration isolation performance and electrical output under different excitation frequencies, the comparison of time histories between the absolute displacement of the isolated payload and the base excitation displacement, the displacement time histories of three groups of piezoelectric cantilever beam oscillators with different parameters (each group containing two oscillators with the same parameters), and the voltage time history curves. The base acceleration amplitude is 0.5 g, the damping ratio *ζ* is 0.05, the load resistance is 450 kΩ, and other parameters are detailed in Table 1. As can be seen from the figure, when the frequency is 1.5 Hz, the payload response amplitude is larger than the excitation amplitude, and the vibration isolation structure has not yet exerted its vibration isolation effect at this time. When the frequency rises to 2 Hz, the payload amplitude is significantly attenuated compared with the excitation amplitude, with a vibration attenuation rate of 69.3% (*T_d_* = 0.307). As the excitation frequency continues to increase, the vibration isolation effect gradually strengthens, reaching 92.6% (*T_d_* = 0.074) at 5 Hz and 98.6% (*T_d_* = 0.014) at 25 Hz. Regarding electrical output, it is worth noting that, when the frequency is 12 Hz and 15 Hz, they coincide with the resonant frequencies of the third and second groups of piezoelectric oscillators, respectively. The corresponding displacement of oscillators in the respective groups is significantly larger than that of the other two groups, and the voltage output is also significantly enhanced. In non-resonant frequency regions, the three groups of piezoelectric oscillators contribute equally to the voltage output.

**Figure 14:**
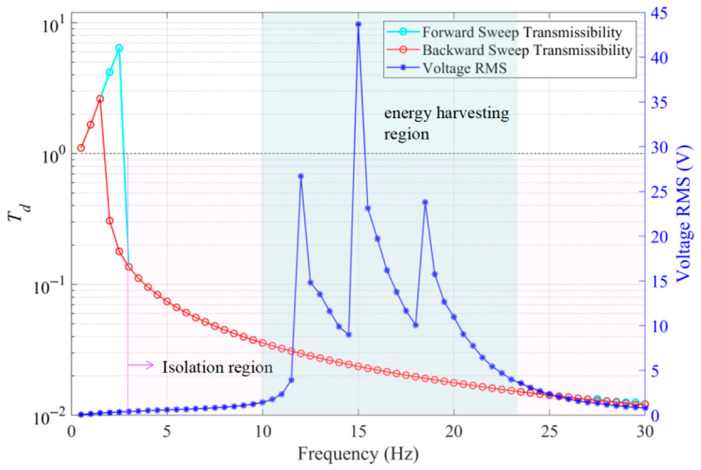
Absolute displacement transmissibility (forward and reverse frequency sweep) and effective value of output voltage varying with excitation frequency (*Z_e_* = 0.5 g; *ζ* = 0.05; *R* = 450 kΩ).

Figure 14 illustrates the variation curves of absolute displacement transmissibility and the RMS value of output voltage with excitation frequency. Within the range of 1.5 Hz to 3 Hz, affected by the system’s nonlinear hardening characteristics, the transmissibility curve exhibits a jump phenomenon. From an engineering standpoint, this is detrimental to maximizing vibration isolation performance in the ultra-low frequency region. Controlling the system’s response to lie on the lower solution branch would facilitate broadening the effective vibration isolation frequency band and enhancing isolation performance in this range. When the excitation frequency exceeds 3 Hz, the vibration isolation performance stabilizes, with the vibration attenuation rate remaining above 86.4%; beyond 10 Hz, it stays above 96%. The system’s effective voltage displays three distinct resonance peaks with varying excitation frequencies, corresponding to the natural frequencies of three groups of piezoelectric oscillators with different parameters. This demonstrates that the array-type design with diversified parameters can effectively widen the energy-harvesting frequency band. Notably, this energy-harvesting band overlaps with the vibration isolation region, avoiding the trade-off between improving energy-harvesting efficiency and maintaining vibration isolation performance, thus enabling the structure to achieve dual functions.

The current prototype employs only three groups of piezoelectric cantilever beam oscillators with differentiated parameters. Through the discrete arrangement of their natural frequencies, it initially achieves the broadening of the energy-harvesting band and its overlap with the vibration isolation region. If the number of oscillator groups is further increased (e.g., to 5–7 groups) and the frequency distribution is optimized, the energy-harvesting resonance peaks can more closely cover the vibration isolation interval. This would systematically expand the frequency range where the “vibration isolation-energy harvesting” dual functions operate synchronously, increase their overlap ratio, and enhance the system’s adaptability to complex excitation environments.

**Figure 15:**
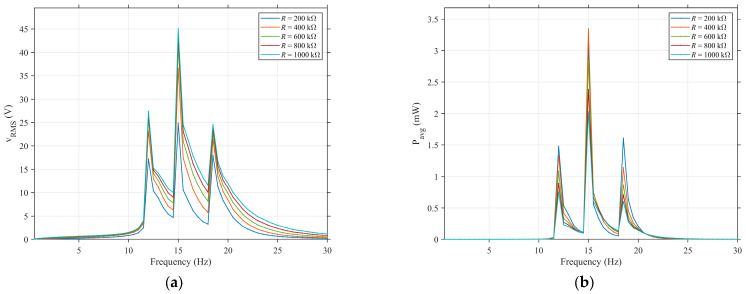
Comparison of effective values of output voltage and average power curves varying with frequency under different load resistances (*Z_e_* = 0.5 g; *ζ* = 0.05): (**a**) effective value of output voltage and (**b**) average power.

Figure 15 illustrates the variation laws of the effective value of output voltage (Figure 15a) and the average power (Figure 15b) with the excitation frequency under different load resistances. It can be observed that changes in load resistance exert no influence on the effective energy-harvesting frequency range. As the load resistance increases, the voltage amplitude at the resonance peak increases monotonically, because a larger resistance can more easily extract the induced voltage of the piezoelectric ceramic. The frequency characteristic of the average power, however, shows distinct behaviors. It is apparent that the power output is maximized when the second group of piezoelectric oscillators resonates; under a 450 kΩ load, the power peak is significantly higher than those with other resistances. For the first and third power peaks (corresponding to the resonance of the third and first groups of piezoelectric oscillators, respectively), the best performance is achieved under a 200 kΩ load. Overall, 400 kΩ is close to the system’s “optimal load resistance”, at which the electromechanical energy conversion efficiency reaches the highest level.

## 5. Conclusions

This paper proposes a dual-function structure integrating low-frequency vibration isolation and broadband energy harvesting. The structure comprises two key components: Component 1 forms a quasi-zero stiffness (QZS) structure by combining a linkage-horizontal spring negative stiffness structure with a vertical spring. Component 2 is an array-type piezoelectric cantilever beam oscillator designed with differentiated parameters. Based on the statics analysis of the QZS structure, a multi-objective function is constructed under the Pareto optimization principle, aiming to minimize the average dynamic stiffness and maximize the zero-stiffness range. The grid search method is employed to obtain the optimal parameter combination. The entire system is modeled as a multi-degree-of-freedom system, and the electromechanical coupling equations are derived. The dynamic response is analytically solved using the harmonic balance method (HBM), and expressions for displacement transmissibility and output power are derived, which are verified by Runge–Kutta numerical simulations. Quantitative analyses are conducted on the influence laws of external excitation conditions and system parameters on vibration isolation and energy-harvesting performance.

The analysis shows that, in terms of vibration isolation performance, the optimized QZS structure exhibits excellent vibration isolation performance, with an initial vibration isolation frequency below 2 Hz. In the low-frequency ranging from 3 to 5 Hz, the vibration isolation rate exceeds 60%, and the minimum transmissibility can reach the order of 10^−2^ (vibration isolation rate > 98%). The QZS structure exhibits hardening characteristics; an increase in excitation amplitude intensifies nonlinearity, leading to an increase in the frequency corresponding to the initial vibration isolation, which is unfavorable for ultra-low-frequency vibration isolation. Reduced damping can improve vibration isolation performance but intensifies the instability of the low-frequency multi-solution region, so moderate damping is more optimal.

In terms of energy-harvesting performance, the array-type piezoelectric cantilever beam energy-harvesting structure with multiple groups of differentiated parameters can effectively broaden the energy-harvesting frequency band, and the energy-harvesting region is located within the vibration isolation region, realizing dual-function synergy. Adjusting the equivalent mass and stiffness of each group of piezoelectric cantilever beams can regulate the position of the energy-harvesting region. The load resistance has a significant impact on the output voltage and average power, and there exists an optimal matching resistance in the system, corresponding to the maximum output power.

It should be noted that this study has certain limitations: For simplicity in analysis, damping was simplified to linear viscous damping during modeling. However, in practice, damping such as slider friction and piezoelectric hysteresis exhibits nonlinear characteristics, which may affect the accuracy of dynamic response analysis. The current conclusions are based on theory and simulation; although the rationality of the model has been verified through the consistency between analytical and numerical solutions, experimental validation is lacking. In subsequent work, a nonlinear damping model will be introduced and optimized via parameter identification, and experimental validation will be conducted (including testing displacement transmissibility and piezoelectric output characteristics, etc.) to provide more reliable support for engineering applications.

## Figures and Tables

**Table 1:** Relevant parameters of the prototype.

Symbol	Meaning	Value
*m*	Mass of the payload (including the platform)	50 kg
*a*	Length of the linkage	0.15 m
*L*	Distance between the payload and the constraint surfaces on both sides	0.194 m
*H*	Distance from the loading platform to the static equilibrium position under no-load condition	0.1166 m
*K_v_*	Stiffness of the vertical spring	24,525 N/m
*L_h_*_0_	Original length of the horizontal spring	0.1 m
*K_h_*	Stiffness of the horizontal spring	32,947 N/m
*ζ*	Damping ratio	0.05
*G*	Number of parallel piezoelectric oscillator groups	3
*n_g_*	Number of piezoelectric oscillators per group	2
*ρ*	Density of the cantilever beam substrate	7850 kg/m^3^
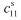	Elastic stiffness coefficient of the substrate	37 GPa
*L_b_*_1,2,3_	Length of the cantilever beam	0.1 m, 0.11 m, 0.12 m
*m_t_*_1,2,3_	Tip mass of the beam	0.08 kg, 0.12 kg, 0.13 kg
*L_p_*_1,2,3_	Length of the piezoelectric layer	0.06 m, 0.07 m, 0.08 m
*b*	Width of the beam and piezoelectric layer	0.02 m
*h_b_*	Thickness of the cantilever beam	0.003 m
*h_p_*	Thickness of the piezoelectric layer	0.0005 m
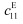	Stiffness of the piezoelectric layer under zero electric field	1.26 × 10^11^ Pa
*e*_31_	Piezoelectric coefficient	−5.2 C/m^2^
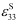	Dielectric constant	3.0 × 10^−8^ F/m
*Θ*_1,2,3_	Electromechanical coupling coefficient	−0.000900 C/m, −0.000945 C/m, −0.000980 C/m
*C_p_*_1,2,3_	Piezoelectric capacitance	7.2 × 10^−9^ F, 8.4 × 10^−9^ F, 9.6 × 10^−9^ F

## Data Availability

No new data were created.
